# High Precision Cervical Precancerous Lesion Classification Method Based on ConvNeXt

**DOI:** 10.3390/bioengineering10121424

**Published:** 2023-12-15

**Authors:** Jing Tang, Ting Zhang, Zeyu Gong, Xianjun Huang

**Affiliations:** 1 State Key Laboratory of Intelligent Manufacturing Equipment and Technology, School of Mechanical Science and Engineering, Huazhong University of Science and Technology, Wuhan 430074, China; j_tang@hust.edu.cn; 2 MOE Key Laboratory of Molecular Biophysics, College of Life Science and Technology, Huazhong University of Science and Technology, Wuhan 430074, China; t_zhang@hust.edu.cn; 3 School of Computer Science and Engineering, Guangzhou Institute of Science and Technology, Guangzhou 510006, China; peterhuang0323@outlook.com

**Keywords:** deep learning, cervical cancer screening, liquid-based cytology

## Abstract

Traditional cervical cancer diagnosis mainly relies on human papillomavirus (HPV) concentration testing. Considering that HPV concentrations vary from individual to individual and fluctuate over time, this method requires multiple tests, leading to high costs. Recently, some scholars have focused on the method of cervical cytology for diagnosis. However, cervical cancer cells have complex textural characteristics and small differences between different cell subtypes, which brings great challenges for high-precision screening of cervical cancer. In this paper, we propose a high-precision cervical cancer precancerous lesion screening classification method based on ConvNeXt, utilizing self-supervised data augmentation and ensemble learning strategies to achieve cervical cancer cell feature extraction and inter-class discrimination, respectively. We used the Deep Cervical Cytological Levels (DCCL) dataset, which includes 1167 cervical cytology specimens from participants aged 32 to 67, for algorithm training and validation. We tested our method on the DCCL dataset, and the final classification accuracy was 8.85% higher than that of previous advanced models, which means that our method has significant advantages compared to other advanced methods.

## 1. Introduction

Cervical cancer is the fourth most common cancer and the fourth leading cause of death in women [1,2,3]. Approximately 600,000 new cases of cervical cancer are diagnosed each year, and over half of cervical cancer patients die as a result [3]. Fortunately, cervical cancer typically develops slowly over time, and early screening can lead to early diagnosis and control of cervical cancer lesions.

The mainstream method for the detection of cervical cancer and its precancerous lesions is based on a high-risk human papillomavirus (HR-HPV) concentration, as HR-HPV concentration changes significantly during the course of having cervical cancer [4,5]. In order to fully explore the variation in HPV concentration in different individuals, scholars have carried out in-depth studies. Zhao et al. [6] conducted a large number of experiments to explore the role of HR- HPV E6/E7 massage RNA (mRNA) in detecting high-grade cervical intraepithelial neoplasia in cervical cancer screening. Shen et al. [7], on the other hand, investigated the role of high-risk human papillomavirus (HPV) in cervical cancer screening in women of different ages. Latsuzbaia et al. [8] established VALHUDES to evaluate the clinical accuracy of HPV assays, to detect cervical precancer in first- to second-phase cervical cancer screening.

Unlike the above HR-HPV concentration-based methods, Vink et al. [9] proposed the use of FAM19A4/miR124-2 methylation analysis for the detection of cervical cancer and its precancerous lesions. Liu et al. [10] proposed the use of liquid-based cytology for the diagnosis of precancerous and cancerous cervical intraepithelial neoplasia (CIN). Bhatla et al. [11] studied screening through HPV testing, cytology, and visual inspection after application of acetic acid (VIA), based on varied resourcing and management of screen-positive lesions, among other methods.Notably, in a follow-up, Liu et al. [12] suggested that HR-HPV-positive but cytology-negative cervical cancer screening results are not uncommon. They intend to investigate the accuracy and diagnostic value of colposcopy for cytology-negative and HR-HPV-positive screening results. Many subsequent studies by scholars have also focused on cytology-based diagnosis of cervical cancer.

In fact, when patients are infected with human papillomavirus (HPV), the cervical epithelial cells undergo various morphological changes, including decreased maturation and abnormal proliferation of squamous epithelial cells [13]. This process is referred to as dysplasia, characterized by loss of squamous cell polarity, nuclear enlargement, increased nuclear-to-cytoplasmic ratio, hyperchromasia, and nuclear condensation [14,15]. These phenomena often indicate a higher possibility of cervical cancer progression [16]. Based on this principle, some researchers have used cytological analysis of cervical scrapings obtained through cell brushing to identify abnormal cervical cells, thereby achieving reliable cervical cancer diagnosis [11,14]. Unfortunately, at present, there is a lack of doctors and diagnostic experience among cervical cytology readers, making it difficult to meet the screening demand for cervical cancer [17]. Additionally, the accuracy of cytological examination may vary due to differences in individual pathologists’ experience. Researchers are actively developing new technologies to achieve more accurate and faster automated diagnosis of cervical cancer [10].

Deep learning is a feature learning method that uses non-linear models and can transform raw data into higher-level and more abstract representations [18]. Since its introduction [19], it has demonstrated powerful capabilities in areas such as speech recognition, image recognition, and natural language processing. In recent years, an increasing number of scholars have become dedicated to exploring the application of deep learning in solving medical problems [20,21,22]. Convolutional neural networks (CNNs) are a type of deep neural network with convolutional structures, exhibiting excellent performance [23,24]. From skin cancer [25] to retinal diseases [26], from tissue pathology image classification [27] to tumor metastasis detection [28], CNNs have seen remarkable achievements in the field of medical image analysis [29].

Some scholars have incorporated CNN technology into the construction of diagnostic models for cervical cancer lesion cells. They built convolutional neural networks with only a few layers. Initially, the models were pretrained on the ImageNet dataset, and then fine-tuned using the HEMLBC dataset [30], which is based on liquid-based cytology techniques, resulting in good performance. Later, Pramanik [31] proposed an ensemble learning method based on the Inception V3 [32] and MobileNet V2 [33] models, also achieving satisfactory results. Building upon this approach, Basak and colleagues proposed a method that combines more models. Specifically, they used models such as Inception V3, VGG-16 [34], ResNet-50 [35], and DenseNet101 [36] for feature extraction, ultimately achieving more robust diagnostic outcomes. Unfortunately, they used the Herlev dataset [37] and the SIPaKMeD dataset [38] for training and validation. These datasets significantly limit the performance of the latest disease classification algorithms, as these existing datasets have limited variation in disease types, cell morphology, and background noise. Therefore, it is necessary to train high-accuracy classification models on challenging datasets, to promote the future clinical application of cervical cytology analysis.

In our work, we adopted a CNN-based approach to develop a cervical cancer diagnostic method. To enhance the reliability of our findings, we rigorously validated our classification results using the highly respected NCCL dataset [15]. Overall, we propose a cervical cancer diagnostic method based on ConvNeXt [39]. In terms of image feature extraction, the ConvNeXt module is currently the best feature extractor, capable of obtaining more abundant feature information. However, in the field of early screening for cervical cancer, due to the redundancy of cell image features and small spatial distances between different cell features, it is difficult to directly use ConvNeXt for cell classification. To address these issues, we first enhance cervical cancer data through self-supervised data augmentation. Subsequently, we utilize the ConvNeXt-based convolutional neural network to extract comprehensive enhanced cervical cancer image features. Finally, we input the extracted features into a random forest algorithm for ensemble learning, resulting in the final outcome. Our ConvNeXt method can effectively classify four cell lines: Negative for intraepithelial lesion or malignancy (NILM); ASC-US&LSIL—atypical squamous cells of undetermined significance (ASC-US), low squamous intraepithelial lesion (LSIL); ASC-H&HSIL—atypical squamous cell/cannot exclude HSIL (ASC-H) and high squamous intraepithelial lesion (HSIL); and SCC&AdC, which mainly includes two types—squamous cell carcinoma (SCC) and adenocarcinoma (AdC), demonstrating potential for automatic early detection of cervical cancer.

The contributions of this work are summarized as follows:

1. We introduced ConvNeXt into the screening field for cervical precancerous lesions and designed a new pipeline to classify cervical cancer cells according to the visual characteristics of cervical cancer cells, which finally achieved a good clinical diagnosis effect;

2. We propose self-supervised data augmentation to augment the data, achieving a comprehensive understanding of cervical cancer image features without increasing the workload with additional data annotation;

3. We propose a method based on a random forest for ensemble learning in the model, effectively improving the model’s ability to identify different subtypes of cervical cancer images;

4. We performed multiple experiments on real large-scale datasets, and the results showed that our cervical cancer cell classification model had a huge accuracy advantage over the previous classification models.

## 2. Related Works

In this section, we first provide an in-depth analysis of the current basic methods of cervical cancer diagnosis, and then explore deep learning models for cervical cytology analysis.

### 2.1. Cervical Cancer Diagnosis

Mainstream screening for cervical cancer relies on several typical diagnostic methods: HPV concentration testing [6,7,8], colposcopy and biopsy [40,41,42], and cytology or PAP smear testing [43,44,45]. Among these, HPV detection refers to the use of changes in HPV concentration in patients to diagnose cervical cancer. This method is one of the most well-used diagnostic methods, but because the HPV concentration in different patients may not maintain a high degree of consistency [46,47], this method requires screening several times to determine the final result. Some scholars have also tried to use other characteristics of patients to make a diagnosis [40]. In recent years, colposcopy has been widely used in developing countries, due to underfunding of health care and the scarcity of cervical cancer screening facilities in low and middle-income countries. During colposcopy, the appearance of the pathological area determines whether the patient has overcome the lesion. These abnormal areas include an acetyl-white area, abnormal vascularization area, mosaic area, and puncture [41]. Based on this basic idea, Adweb et al. [42] proposed a classification method based on a VGG network. Meanwhile, Xu et al. [43] proposed a method based on a multi-branch CNN.

Given that colposcopy is not as reliable as PAP tests and that HPV tests are too expensive, some studies have focused on PAP tests. Soni et al. [44] proposed an auxiliary diagnosis and treatment method based on CNN-CRF, which achieved good results. Fang et al. [45] continued this line of thinking. They used the feature representations learned from multiple nuclei of different sizes to construct a deep convolutional neural network for diagnosis. Considering the limitations of a single model, Mohammed et al. [46] detected abnormal cervical cells using an ANN classifier and features extracted by VGG-16 and Googlenet, and achieved good results. Kavitha et al. [47] and Attallah et al. [48] also followed this basic line of thought.

### 2.2. Deep Learning Models for Cervical Cytology Analysis

In recent years, deep learning has been widely used in assistive systems for identifying types of cervical cancer cells. Starting from the basic idea of model construction, we divided the current deep learning models for cervical cytology analysis into two categories according to their target use: (i) constructing diagnostic models based on the idea of cytoplasm and nucleus segmentation; (ii) constructing a diagnostic model based on the idea of pathological cell classification.

**Segmentation of pathological cells.**

A common cytological auxiliary screening method for cervical cancer is to promote the detection and grading of cervical cancer using graph-based methods based on the segmentation results of complex non-convex regions [49]. Bnouni et al. [50] proposed a collection preconditioning method to realize the segmentation of cervical cancer cells based on a CNN. Subsequent scholars have continued this idea, and Sellamuthu et al. [51] proposed an improved deep learning algorithm based on a double-tree complex wavelet transform (DTCWT). De et al. [52] introduced a mask-region-based CNN method, which also achieved good segmentation performance. Wita et al. [53] proceeded from the perspective of features. They integrated MobilenetV2 networks to convert ordinary convolution to deep split convolution, improving the network’s transmission and feature utilization.

**Classification of pathological cells.**

Cervical cancer diagnosis based on the basic idea of classification is the most mainstream AI-assisted diagnosis and treatment method at present and has been studied by many scholars in recent years. Taha et al. [54] used a pretrained CNN architecture as a feature extractor and used the output features as inputs to train a support vector machine classifier. Ghoneim et al. [55] proposed a detection and classification system for cervical cancer cells based on a convolutional neural network (CNN). The cell images were fed into a CNN model to extract deep learning features. Then, an extreme learning machine (ELM)-based classifier classified the input images. Lin et al. [56] proposed a cell classification method based on appearance and morphology, based on a CNN. Differently from the above methods, they studied the classification effect when inputting images from different channels, and finally selected images from five channels as the input for the model. Considering the limitations of a single model, subsequent scholars [57,58,59] introduced the basic idea of model integration and carried out model fusion based on features extracted from multiple CNNS.

In general, most of the existing cell diagnostic models for cervical cancer lesions have been trained and verified based on the Herlev dataset [37]. The Herlev dataset is a cervical cancer image dataset based on Pap smears collected using microscopes and digital cameras. In the Herlev dataset, according to Bethesda’s criteria, cell images are divided into four types: NILM (negative for intraepithelial lesions or malignancies), LSIL, HSIL, and SCC. In addition, some scholars have also conducted model training and verification of low-cost cervical cancer screening based on the CerviSCAN dataset [60] and HEMLBC dataset [30] based on liquid cytology technology. We observed that these datasets greatly limit the performance of the latest disease classification algorithms, because the existing datasets have limited variation in terms of disease type, cell morphology, and background clutter. Therefore, high-precision classification models need to be trained on challenging datasets, to facilitate future clinical applications of cervical cytology analysis.

## 3. Analysis of the DCCL Dataset

Here, we used a large-scale cervical cytology dataset named Deep Cervical Cytological Levels (DCCL) [15] to model a more robust cervical cancer diagnosis system. To our knowledge, this is the largest set of cervical cytology data, and the total data volume is ten times that of the previous benchmark dataset. In order to achieve a better cytological classification, we first performed an in-depth analysis of the dataset. It is worth noting that, in order to ensure the fairness of model comparison, we did not use other datasets for additional training. At the same time, considering the limitations of other datasets, we only carried out the research based on the DCCL dataset.

### 3.1. Dataset Overview

There are 1167 cervical cytological specimens from participants aged 32 to 67 years in the DCCL dataset. These specimens were prepared using the ThinPrep method and stained through Pasteur staining. They were collected by four provincial medical centers from 2016 to 2018. The collected cervical cancer images generated by DCCL included 933 positive patients and 234 normal cases. The image labels of cervical cancer came from the pathological report. All slides were uniformly scanned using one of three digital slide scanners (Nanozoomer2.0HT, KFBIO KF-RPO-400, or AperioAT2), all of which have 200× zoom and 24-bit color.

### 3.2. Dataset Processing

**Figure 1:**
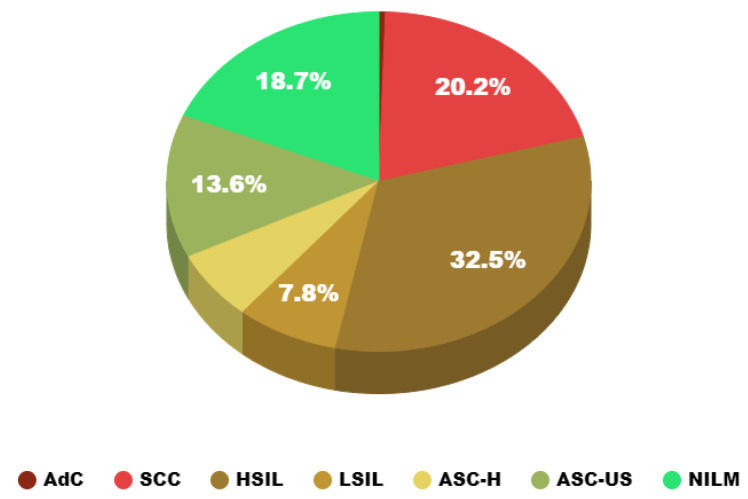
Pie chart of cell distribution.

Each cervical cancer image was trimmed into a grid with a rectangular area of approximately 1200 × 2000 pixels (physical size 1011.6 microns × 606.96 microns). Usually, a picture of cervical cancer is converted into 700–800 color blocks. Specifically, the slide distribution and patch distribution are shown in Table 1. It is worth noting that (i) all data used in our research were strictly anonymous; (ii) the type of slide and patch came from the diagnosis of the pathologist; and there are 34,382 images of cervical cells in the DCCL dataset. The specific data distribution is illustrated in Figure 1.

**Figure 2:**
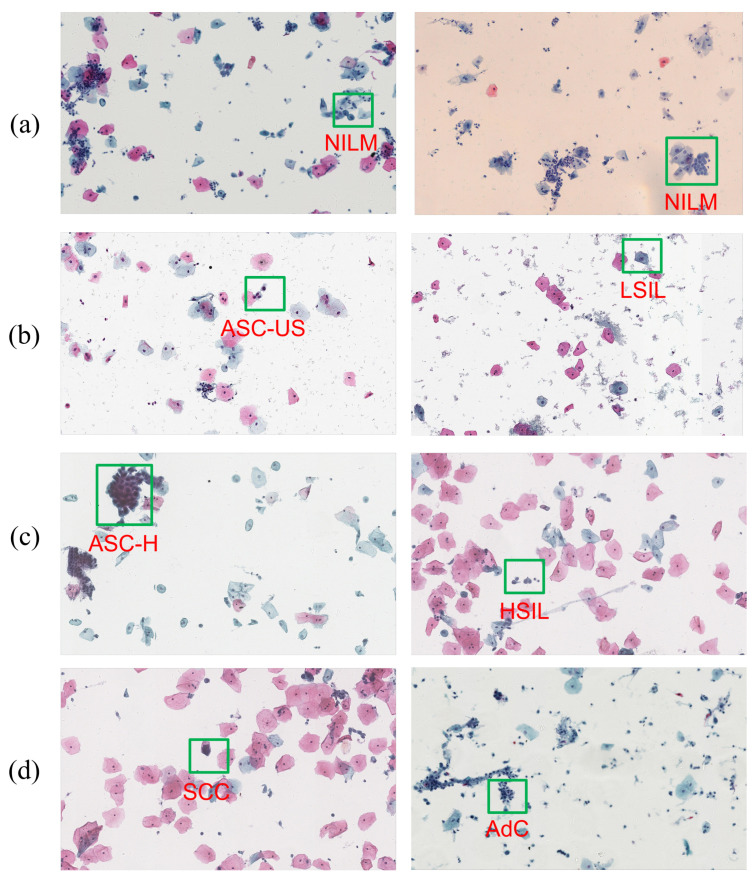
Examples of cells, where (**a**) are negative for intraepithelial lesion or malignancy (NILM) cells; (**b**) are atypical squamous cells of undetermined significance (ASC-US) and low squamous intraepithelial lesion (LSIL) cells; (**c**) are atypical squamous cell/cannot exclude HSIL (ASC-H) and high squamous intraepithelial lesion (HSIL) cells; and (**d**) are squamous cell carcinoma (SCC) and adenocarcinoma (AdC) cells.

Considering some of the inherent challenges in cervical cytology identification, such as intraclass differences (for example, some LSIL cells have clear perinuclear cavities, but the rest do not) and similarities between class differences (for example, HSIL and SCC both have high nuclear-to-cytoplasmic ratios), which can be seen in Figure 2, the dataset divides the images into seven different cell image types, which are divided into three categories: squamous intraepithelial precancerous lesion cells, cancer cells, and cells with negative intraepithelial lesion or malignant tumor (NILM). Among these, squamous intraepithelial precancerous lesion cells are divided into four types with increasing severity: atypical squamous cells with undetermined significance (ASC-US), low-grade squamous intraepithelial lesion (LSIL), atypical squamous cells with high-grade squamous intraepithelial lesion (ASC-H), and high-grade squamous intraepithelial lesion (HSIL). Cancer cells are mainly divided into two types: squamous cell carcinoma (SCC) and adenocarcinoma (AdC). A classification diagram is shown in Figure 2.

**Figure 3:**
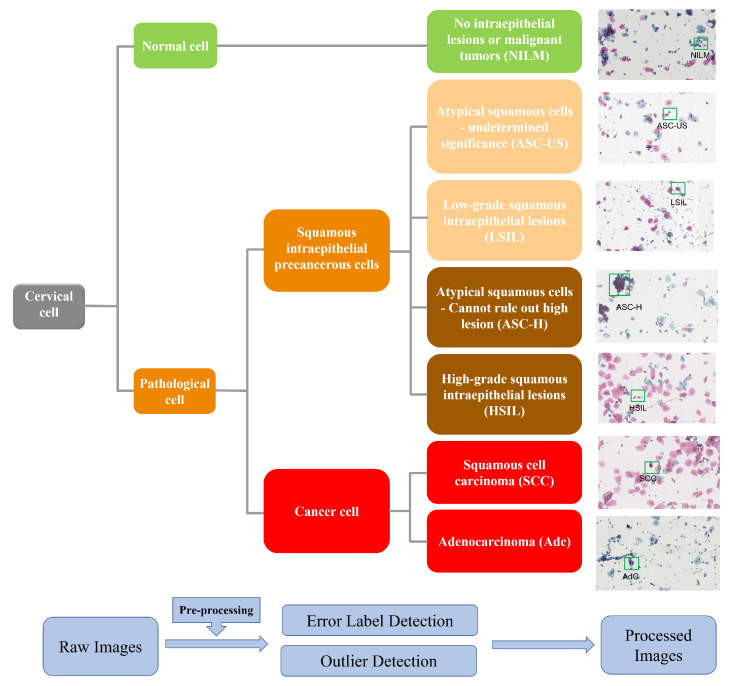
Classification of cervical cancer cells.

In order to ensure the reliability of the experiment, 8619 distinctive cervical cytological images were extracted from the original dataset. All pictures were finally divided into four categories according to the severity of the lesions, and the severity from low to high was NILM, ASC-US&LSIL, ASC-H&HSIL, and SCC&AdC. The classification of pictures of the different types of cells is shown in Figure 3 (the severity increases in turn) and Table 2.

### 3.3. Data Characteristic Analysis

By comparing with other widely used datasets, including CerviSCAN [60], Herlev [37], and HEMLBC [30], we analyzed the attributes of DCCL. Table 3 shows their differences in terms of target task types, data size and diversity, lesion types, and accessibility. Taking task types as an example, CerviSCAN and Herlev are only used for cell type classification, where samples are cropped from the original slides without contextual information. On the other hand, HEMLBC is used for target detection of pathological cells, but it is challenging to build a high-precision pathological cell detection model due to the limited scale of the data. In contrast, DCCL can be used for high-precision cell type classification, enabling reliable diagnosis and analysis of cervical cancer.

We also conducted a cross-comparison of the different attributes of the various datasets, and the comparison results are shown in Table 3. Table 3 reveals that compared to CerviSCAN, DCCL exhibits a greater variety of lesions. Due to the diversity in digital slide scanner types, patient ages, pathological cell types, and background noise, DCCL poses a larger range of challenges. All these different factors are crucial for establishing a robust and reliable clinical application system. Based on such prior understanding of the dataset, we needed to consider how to extract meaningful information from the dataset during the construction of the model, instead of allowing the model to learn from data noise. Additionally, it was necessary to consider building more robust classification models. To address these issues based on our data analysis findings, we proposed a self-supervised data augmentation method and a model ensemble approach specifically tailored to this dataset.

## 4. Methodology

### 4.1. Pipeline

**Figure 4:**
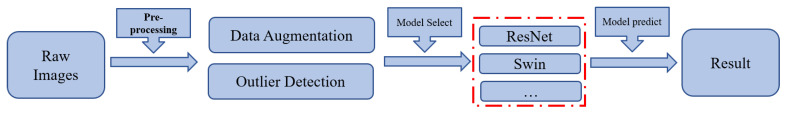
Traditional classification pipeline. The red boxes represent the integration of different models.

**Figure 5:**
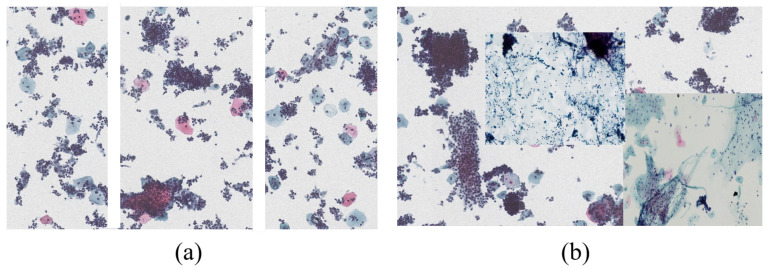
Traditional data augmentation methods. (**a**) shows a method of lengthwise concatenation of images with different labels, while (**b**) shows a method of random concatenation of images with different labels.

The traditional image classification scheme is shown in Figure 4. The first step is to perform data preprocessing, such as data augmentation and outlier detection on the input image. Among these, data augmentation can generate more equivalent data to artificially expand the training dataset in the case of limited data, which is an effective means of overcoming a shortage of training data. At present, this is widely used in medical diagnosis scenarios with insufficient data. The earliest data augmentations included geometric transformations, color transformations, rotations, and affine transformations, among others. Later scholars also proposed ways to mix images with different labels. For humans, the data generated by mixing images seems meaningless due to the lack of interpretability of this method. However, for a model, such as a simple and effective data augmentation algorithm, there are a series of works of related research [61,62]. In Figure 5, (a) shows a method of lengthwise concatenation of images with different labels, while (b) shows a method of random concatenation of images with different labels. The current mainstream data augmentation methods also include AutoAugment [63], based on automatic search for improved data augmentation, and RandAugment [64], which reduces the search space for data augmentation to address the massive computational costs of automatic data augmentation.

After the data preprocessing is completed, the processed data and labels are divided into training sets and validation sets. After the training set is input into different models for model training, researchers select a model based on the performance of different models on the verification set, and then build a detection method based on the model. In general, such a pipeline typically involves simple data processing and model selection, training, and prediction based on a single model. However, the datasets for cervical cancer diagnosis have weak feature data, and the traditional scheme based on a single model and simple data augmentation cannot achieve good results.

**Figure 6:**
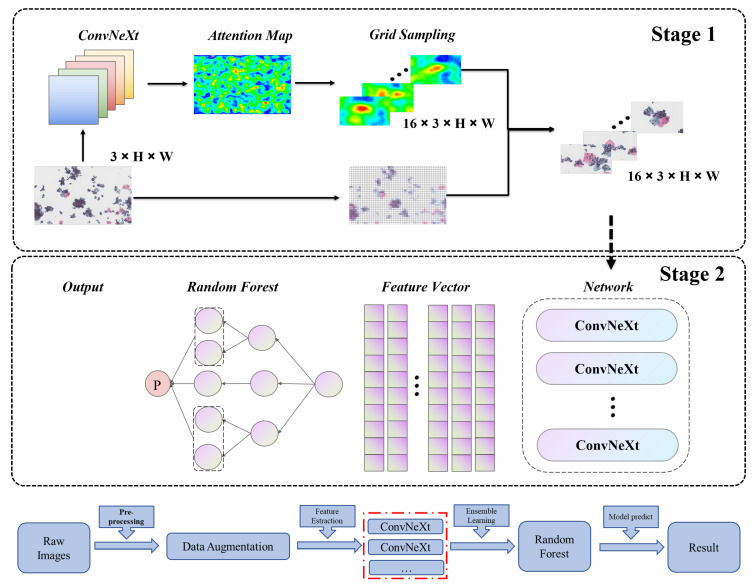
Overall scheme.The red boxes represent the integration of different models.

Here, as shown in Figure 6, we proposed a two-stage cervical cancer cell classification model based on ConvNeXt, which can effectively grasp cell local context and geometric information and is very suitable for cervical cancer cell classification tasks. The feature extraction unit of the original multiscale feature fusion network in ConvNeXt underwent optimization compared with other famous CNN backbones. This involved replacing it with a combination of depth separable convolution, an inverse bottleneck layer, and Gaussian error linearity. Additionally, a larger convolution kernel was employed to capture more abundant feature information. These enhancements resulted in improved regional proposals when input into the regional proposal network. These improvements enable ConvNeXt to achieve advanced performance on many publicly available datasets, enabling other researchers to migrate our model to different medical classification tasks.

The overall process is shown in Figure 6, which was divided into stage 1 and stage 2. In stage 1, we introduced a self-supervised learning method to enhance the data. We use the attention map to determine the possible diseased cells and then segment the image based on the determination results to expand the dataset to 16 times the original, laying the foundation for the construction of the subsequent high-precision classification model. In stage 2, considering the complexity of cell classification tasks and the small differences between different types of cells, the model was very mixed. Here, we introduced an ensemble learning strategy and used a random forest strategy to optimize the final classification results.

### 4.2. Self-Supervised Data Augmentation

**Figure 7:**
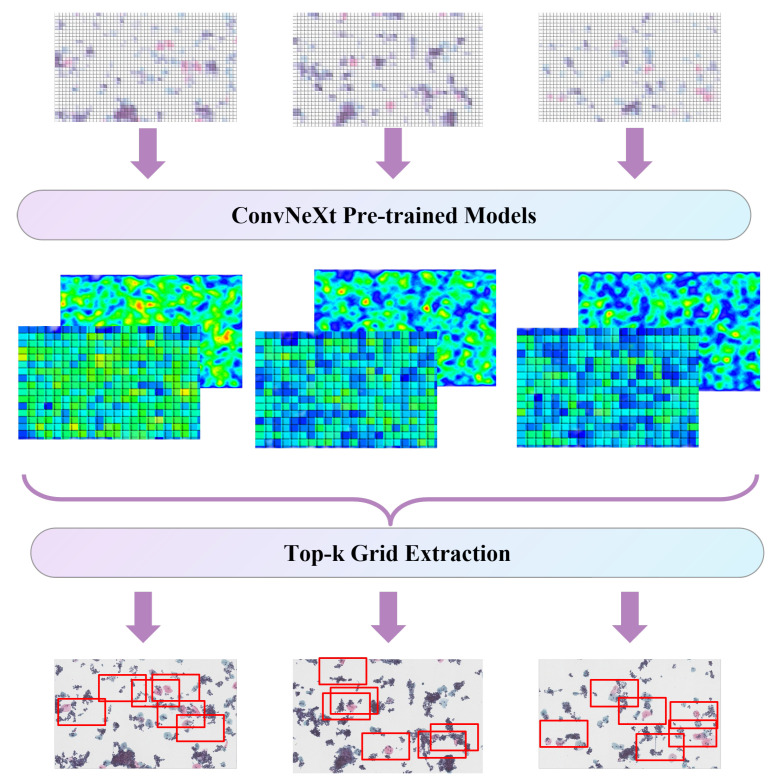
Data augmentation based on the self-supervision method.

A larger dataset means a more accurate classification model. To expand the dataset, we proposed a novel data enhancement method based on a self-monitoring method using an attention map. This method could visualize the attention of ConvNeXt, and data augmentation based on this method made the trained ConvNeXt model pay better attention to the lesion areas in the whole image. We first trained a preliminary classification model based on the original dataset, and then we extracted an attention map of the classification model and decomposed it into m×m(m>4) grids. The specific process is shown in Figure 7.

Regarding the specific extraction method of the attention maps, here we used class-activation heatmaps from the original classification model. Specifically, we refer to the method mentioned in Grad-CAM [65]:

1. Predefine the specific class NLIM;

2. Perform forward calculation to obtain the network output value, $Y_{NLIM}$, corresponding to the specified class NLIM, and perform backward propagation;

2. Extract the feature maps of each channel from the last layer of the ConvNeXt backbone network and calculate the gradients of each channel feature map as new gradient maps;

3. Apply global average pooling (GAP) to the different channel gradient maps to obtain the gradient weight values, $W_{NLIM}$, for each channel;

4. Calculate the weighted average of the feature maps of each channel using $W_{NLIM}$;

5. Apply ReLU activation and upsampling to obtain the target attention map;

Then, we counted the intensity values of attention in m×m grids and selected the 16 grids with the highest intensity using a depth-first search (DFS). The specific DFS strategy was as follows:

(1) Randomly select a visited pixel grid in the first-line pixel grid;

(2) Mark the selected pixel grid as visited;

(3) Sequentially search from the 1,2,3,…,n adjacent pixel grids beneath the pixel grid, which have not been visited;

(4) If there are still unvisited adjacent pixel grids, select the pixel grid with the lowest row as the starting vertex, and go back to step (2);

(5) If all pixel grids have been visited, then finish;

After calculating the 16 maximum attention values and their corresponding positions in the global image, we extracted 16 grids around that position with a size of (H/16) × (W/16). These grids were then resized to the scale of H × W and subsequently input into the network model for training. Here, H and W are the sizes of the image input to the model. Considering that we used the ConvNeXt tiny model to build the classification model, we extracted the cervical cancer cell image with a pixel scale of 768×768 based on the center points of 16 grids and finally completed the 16-fold expansion of the dataset (Figure 2).

### 4.3. Ensemble Learning Strategy

To improve the accuracy of the classification model, we introduced a multi-model fusion strategy. First, we trained the classification network using ten-fold cross-validation to obtain 10 different models. Then, we used the random forest method to fuse the different classification models. Specifically, we took the output of the SoftMax layer of the model as a feature vector, and then input the random forest. Here, we used the sklearn library [66] to build a random forest, in which the number of decision trees in a random forest was set to 120, the Gini coefficient was adopted as the algorithm for the decision tree, the maximum depth of the decision tree was set to 5, and the other parameters were kept as default.

We took the output label of the model as features and built a feature vector *F* with a dimension of 10. The sample size of the dataset was |F| and there were K classes $C_{k}$, $k=1,2,\ldots K.\;\left|C_{k}\right|$ was the number of samples belonging to $C_{k}$ and $\sum_{k=1}^{K}\left|C_{k}\right|=\left|F\right|$. When each node of the decision tree needs to be split, m attributes are randomly selected from these 10 attributes, meeting the condition m<<M. Later, we obtained n subsets $F_{1},F_{2},\ldots ,F_{n}$ of the feature vector $F,\left|F_{i}\right|$ representing the number of feature vectors contained in it. The set of $F_{i}$ belonging to the $C_{k}$ class in the subset was $F_{ik}=F_{i}\cap C_{k}$. We calculated the information gain of the dataset using the following steps:

1. Calculate empirical entropy $H\left(F\right)=-\sum_{k=1}^{K}\frac{\left|C_{k}\right|}{\left|F\right|}\log_{2}\frac{\left|C_{k}\right|}{\left|F\right|}F$;

2. Calculate the empirical conditional entropy of feature *A* pair datasets

$$H\left(F|A\right)=\sum_{i=1}^{n}\frac{\left|F_{i}\right|}{\left|F\right|}H\left(D_{i}\right)=-\sum_{i=1}^{K}\frac{\left|F_{ik}\right|}{\left|F_{i}\right|}\log_{2}\frac{\left|F_{ik}\right|}{\left|F_{i}\right|};$$

3. Calculate the information gain g(F,A)=H(F)−H(F∣A)

Then, we selected one attribute from the m attributes as the split attribute of the node based on the calculated information gain. In the process of building the decision tree, each node should be split in this way until it can no longer be split. Finally, we constructed a large number of decision trees and completed the construction of the random forest model based on these decision trees.

## 5. Experiments

### 5.1. Experiment Setup

Hardware: Here, we used a GPU for training acceleration, the training algorithm was run on Ubuntu 18.04, and the training machine was a 64-bit server with 10 vCPU Intel Xeon Gold 6248R and 512 G memory. The GPU type of the server was a 1*A100-PCIE-40GB (40 GB). In total, our network model training algorithm took about 4 h to run on this hardware configuration.

Metrics: Considering that the classification-based cervical cancer diagnosis method is more robust than the detection-based cervical cancer diagnosis method in actual diagnosis, we used the basic idea of classification to analyze the diagnosis of cervical cancer cells. We verified the progressiveness of the diagnostic model for cervical cancer diagnosis and analysis tasks based on the ConvNeXt model proposed in this paper by comparing with the performance of different published models in this task. We followed the evaluation indicators used in [37] and used the accuracy, precision, recall, and F1-score as the evaluation indicators. Accuracy is the ratio between correctly classified samples and the dataset size. Precision is the ratio of true positive samples (correctly identified as positive) to the total number of samples identified as positive. Recall is the ratio of true positive samples to the total number of actual positive samples. F1-score is a weighted harmonic mean of precision and recall, and is commonly used to evaluate the performance of classification models. Using multiple metrics to evaluate algorithm models allows for a comprehensive understanding of the model’s preferences. Additionally, in the DCCL dataset, the distribution of samples among different classes is imbalanced. A single accuracy metric may not reflect the model performance accurately. By introducing multiple metrics, we could better evaluate the model’s ability to identify different categories of cervical cells. We calculated these using the following equation:

$$\;\mathrm{Accuracy}\;=\frac{TP+TN}{TP+TN+FP+FN}.$$

$$\;\mathrm{Precision}\;=\frac{TP}{TP+FP}.$$

$$\;\mathrm{Recall}\;=\frac{TP}{TP+FN}.$$

$$\;F1-\mathrm{score}\;=\frac{2\times Precision\times Recall}{Precision+Recall}.$$

True positive (TP): an outcome where the model correctly predicts the positive class.

True negative (TN): an outcome where the model correctly predicts the negative class.

False positive (FP): an outcome where the model incorrectly predicts the positive class.

False negative (FN): an outcome where the model incorrectly predicts the negative class.

It is worth noting that when one category is considered positive, the rest of the categories are considered negative.In this way, the accuracy rate and recall rate of each category could be obtained in the multi-classification scenario of this paper. In calculating the accuracy and recall rates for the entire confusion matrix, we averaged the index values for each category.

### 5.2. Fine-Tuning Policy Verification

**Figure 8:**
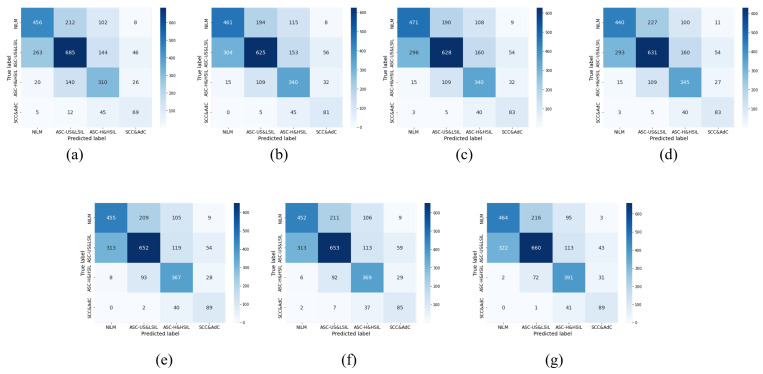
Comparisonof the test set classification accuracy under different optimization strategies: (**a**) Raw ConvNeXt; (**b**) +CutMix; (**c**) +Autoaug; (**d**) +Randaug; (**e**) +SDA; (**f**) +ELS; (**g**) Our Method.

To validate the effectiveness of self-supervised data augmentation (SDA) and ensemble learning strategy (ELS), this study first conducted comparative experiments between the original ConvNeXt method and the ConvNeXt method using SDA and ELS. The experimental results are shown in Table 4 and Figure 8. The ConvNeXt method with SDA and the ConvNeXt method with ELS achieved accuracy improvements of 1.69% and 1.53%, respectively, demonstrating significant progress in the diagnosis and analysis of cervical cancer. Finally, with the combination of SDA and ELS, the accuracy of the ConvNeXt method reached 63.08%, a 3.31% improvement over the original ConvNeXt method. This meets the basic medical requirements for cervical cancer auxiliary reading and can be applied in the field of cervical cancer diagnosis. These results indicate that the proposed enhancement methods provide useful clues for improving cell type classification. Moreover, in terms of precision and recall, the SDA and ELS method proposed in this study also achieved a relatively stable performance improvement. Ultimately, our method achieved an F1-score of 62.82%. We also performed validation tests on other common data augmentation methods. When applying CutMix [62] or Randaug [64] on this dataset, the accuracy and precision could even decrease. Autoaug [63] showed a slight improvement in performance, but the magnitude of improvement was minimal. This was because the DCCL dataset used in this study has complex data features and small inter-class differences, causing these traditional data augmentation methods to become ineffective. These verification results further demonstrate the advancement of the proposed method.

### 5.3. Comparison with Advanced Methods

**Figure 9:**
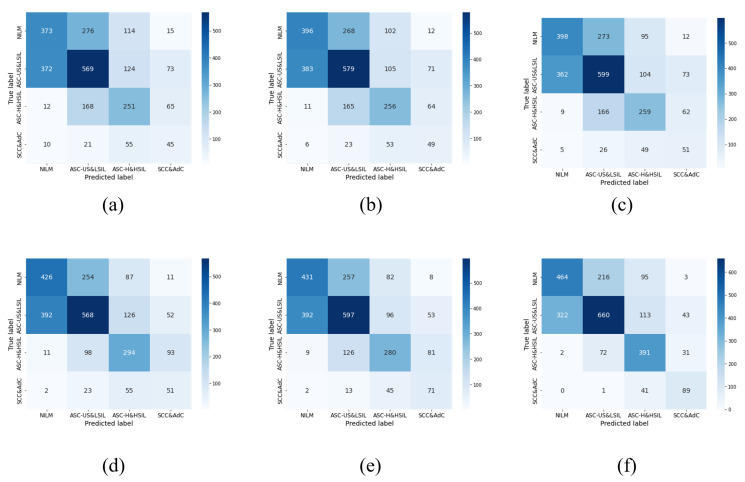
Comparison of the classification accuracy of the test sets using different advanced methods: (**a**) ResNet [35]; (**b**) Inception [32]; (**c**) DenseNet [36]; (**d**) Swin [67]; (**e**) Beit [68]; (**f**) Our Method.

To validate the advancement of the proposed approach in this study, we compared it with several classical algorithms and existing state-of-the-art algorithms. The final experimental results are shown in Table 5 and Figure 9. The method proposed in this study achieved a good performance. Compared to traditional convolutional neural network models such as Inception-v3 [32], ResNet-101 [35], DenseNet-121 [36], which were early models used for classification tasks, the approach proposed in this study showed significant improvements in terms of accuracy, precision, and recall. Additionally, compared to the current mainstream network models, this approach also demonstrated a substantial improvement in accuracy. In comparison with the classification method based on a Swin transformer [67], our approach achieved a 10.43% improvement. Furthermore, compared to the classification method based on Beit [68], our approach achieved an 8.85% improvement.

## 6. Conclusions

This paper aimed to build a high-precision cervical cell classification model based on the large DCCL cervical cancer cell benchmark dataset. This model is intended to contribute to future research and clinical studies in cervical cancer screening. The dataset poses inherent challenges in cervical cell identification, such as intra-class variations (e.g., some LSIL cells having clear perinuclear halos, while others do not) and inter-class similarities (e.g., HSIL and SCC both having high nucleus-to-cytoplasm ratios), which are commonly encountered in clinical settings. To achieve high-precision cervical cancer diagnostic analysis on the DCCL dataset, we introduced a high-performance network named ConvNeXt as the backbone of a neural network model. We proposed a novel self-supervised data augmentation technique for data enhancement, as well as an ensemble learning strategy based on random forests for model enhancement. We conducted extensive experiments on the DCCL dataset to demonstrate the effectiveness of our approach. An ablation study involving different augmentation methods showcased the effectiveness of our proposed data augmentation and model enhancement schemes. The comparative experimental results with different state-of-the-art models indicated that our model outperformed the others in various performance evaluation metrics. This suggests that our approach can greatly assist in real cervical cancer diagnosis processes. In future research, we will also further optimize our data augmentation methods, perform more in-depth studies on cell integrity, and conduct more tests on the interpretability of the model.

## Figures and Tables

**Table 1:** Statistics of pictures and patches by type.

Cell Type	Train	Val	Test	Total
NILM	2588	1540	2292	6420
ASC-US	2471	838	1378	4687
ASC-H	1147	543	591	2281
LSIL	1739	346	595	2680
HSIL	5890	1807	3482	11,179
SCC	3006	1225	2731	6962
AdC	122	20	31	173
Total	16,963	6319	11,100	34,382

**Table 2:** Attributes of the processed cervical cancer cell dataset.

Cell Type	Train	Val	Test	Total
NILM	1046	494	778	2318
ASC-US&LSIL	2108	731	1138	3977
ASC-H&HSIL	992	401	496	1889
SCC&AdC	243	61	131	435
Total	4389	1687	2543	8619

**Table 3:** Attribute comparison among the different cervical cancer cell datasets.

Dataset	Patients	Labelled Patches	Labelled Cells	Lesion Cell Types	Classification Annotations	Detection Annotations	Open Source
CerviSCAN [60]	82	900	12,043	3	✓	**×**	✓
Herlev [37]	-	-	917	3	✓	**×**	✓
HEMLBC [30]	200	-	2370	4	✓	✓	**×**
DCCL [15]	1167	14,432	34,392	6	✓	✓	✓

**Table 4:** Comparison of test set classification accuracy under different optimization strategies.

Method	Accuracy (%)	Precision (%)	Recall (%)	F1-Score (%)
Raw ConvNeXt	59.77	56.12	58.49	57.09
+CutMix [62]	59.26	55.98	61.14	57.83
+Autoaug [63]	59.85	56.62	61.91	58.53
+Randaug [64]	58.95	56.11	61.23	58.02
+SDA	61.46	58.61	64.43	60.80
+ELS	61.30	58.01	63.69	60.15
Our Method	63.08	60.78	66.10	62.82

**Table 5:** Comparison of the classification accuracy of the test sets under different advanced methods.

Method	Accuracy (%)	Precision (%)	Recall (%)	F1-Score (%)
ResNet [35]	48.68	43.13	45.72	44.08
Inception [32]	50.33	45.08	47.70	46.04
DenseNet [36]	51.39	46.14	48.74	47.09
Swin [67]	52.65	47.11	50.72	48.32
Beit [68]	54.23	50.20	54.63	51.71
Our Method	63.08	60.78	66.10	62.82

## Data Availability

The data presented in this study are not publicly available due to ongoing research in this field.
